# Hypoxia-Induced Extracellular Vesicles Derived from Human Umbilical Cord Mesenchymal Stem Cells Regulate Macrophage Polarization and Enhance Angiogenesis to Promote Diabetic Wound Healing

**DOI:** 10.3390/biom15111504

**Published:** 2025-10-24

**Authors:** Yongfeng Su, Junda Lu, Feiyuan Liang, Jianwen Cheng

**Affiliations:** 1 Department of Orthopaedics Trauma and Hand Surgery, The First Affiliated Hospital of Guangxi Medical University, Nanning 530021, China; 202310045@sr.gxmu.edu.cn (Y.S.); 202320201@sr.gxmu.edu.cn (J.L.); 202220255@sr.gxmu.edu.cn (F.L.); 2 Guangxi Clinical Medical Research Center for Orthopedic Disease, The First Affiliated Hospital of Guangxi Medical University, Nanning 530021, China

**Keywords:** diabetic wounds, hypoxia, extracellular vesicles, angiogenesis, oxidative stress

## Abstract

**Background**: Diabetic wound healing has always been a clinical challenge with minimal response or efficacy to standard treatment. This study aims to assess the therapeutic potential of hypoxia-induced extracellular vesicles (hy-EVs) produced by human umbilical cord mesenchymal stem cells (HUCMSCs) to treat diabetic wounds. **Methods**: HUCMSCs were isolated from umbilical cord tissue, cultured under hypoxic conditions to induce the release of extracellular vesicles (EVs) and compared with normoxia-induced extracellular vesicles (n-EVs). We assessed the functions of hy-EVs on human skin fibroblasts (HSFs) and human umbilical vein endothelial cells (HUVECs) in vitro. Simultaneously, we analyzed the pro-angiogenic effects of hy-EVs, their effects on macrophage polarization, and their ability to scavenge endogenous reactive oxygen species (ROS). In addition, a diabetic wound model was established to assess the curative effect of hy-EVs in diabetic wound healing. **Results**: We found by in vitro study that hy-EVs markedly improved the functional activities of HSFs, thus significantly promoting wound repair. Remarkably, it was determined that hy-EVs greatly enhanced the proliferation and migration ability as well as the angiogenic ability of HUVECs, while promoting the expression of hypoxia-inducible factor-1α (HIF-1α), vascular endothelial-generation-associated factor A (VEGFA), and platelet endothelial adhesion molecule (CD31), which suggested that hy-EVs can effectively activate the HIF-1α pathway to promote angiogenesis. Above all, we found that hy-EVs promoted the expression of CD206 while decreasing the expression of CD86, suggesting that hy-EVs could induce macrophages to shift from M1-type (pro-inflammatory) to M2-type (anti-inflammatory), thereby modulating the inflammatory response. Additionally, hy-EVs inhibited ROS production in both HSFs and HUVECs to reduce oxidative stress. In vivo results showed that hy-EVs enhanced collagen deposition and angiogenesis, modulated macrophage polarization, and inhibited immune response at the wound spot, which significantly enhanced diabetic wound healing. **Conclusions**: Our study shows that hy-EVs significantly promote angiogenesis through activation of the HIF-1α pathway, modulate macrophage polarization and attenuate cellular oxidative stress, possibly through delivery of specific miRNAs and proteins. Our discoveries offer a key theoretical basis and potential application to develop novel therapeutic strategies against diabetes-related tissue injury.

## 1. Introduction

Diabetes is a disorder of metabolism that causes hyperglycemia. There has been a growing trend in diabetes occurrence, thereby causing a huge burden on the public health systems throughout the world [1,2]. Diabetic patients suffer from multiple complications, among which diabetic wounds are a major concern [3]. The main features of diabetic wounds include wound inflammation, impaired angiogenesis, and excessive oxidative stress, globally affecting almost 6.3% of the diabetic population each year [4,5]. Failure of diabetic wound healing significantly impairs patients’ quality of life. Moreover, these wounds also result in possible infections, gangrene and other issues, resulting in severe chronic wounds, the final outcome of chronic wounds is often amputation and/or death [6,7]. Although several treatment options are available for diabetic wounds, including glycemic control, dressing changes, and antimicrobials, as well as debridement, their overall success rate is quite low [8,9,10]. Thus, the finding of innovative therapeutic options that can potently enhance diabetic wound repair has become an urgent need.

HUCMSCs are a unique type of pluripotent stem cells obtained from neonatal umbilical cord tissue; these cells have self-renewal ability, multidirectional differentiation potential and remarkable immunomodulatory properties [11]. They have attracted considerable attention owing to their abundant sources in recent years, low immunogenicity, and the absence of ethical controversies in their acquisition and usage, so they have been widely employed within the realm of regenerative medicine [12]. HUCMSCs possess the capacity to release a range of biologically active molecules, including growth factors, which is essential to promote regenerative tissue repair [13]. Recent studies have revealed that HUCMSCs predominantly facilitate tissue repair via paracrine mechanisms [14]. Notably, the EVs secreted during this process serve as crucial mediators in intercellular communication, playing an indispensable role in cellular interactions [15]. These EVs derived from HUCMSCs are rich in diverse bioactive molecules, including proteins, lipids, and RNA [16]. Especially, compared to their parental cells, EVs offer advantages such as superior tissue compatibility, higher stability, and reduced immunogenicity [17]. Studies suggest that EVs have a wide range of applications in different fields, including disease diagnosis, treatment and prognosis; they show excellent potential in skin wound healing in particular [18,19]. For example, a recent study found that MSC-derived exosomes enhanced skin wound healing by inhibiting AIF nuclear translocation and attenuating cell death, and another study found that MSC-derived exosomes promoted M2 polarization and enhanced skin wound healing [20,21].

Although EVs show some potential for skin wound healing, the main barriers to their commercial production and clinical translation are their low bioactivity and yield, and their limited effectiveness in complex chronic diseases such as diabetic wounds [22]. Therefore, there is a need to develop suitable methods to enhance the activity and increase the yield of EVs. Hypoxia induction is a significant intervention, which can change the biological characteristics and functions of mesenchymal stem cells by influencing their biological behavior and functions, thereby altering the secretion of EVs [23]. The positive effects of hypoxic conditions on mesenchymal stem cells (MSCs) may stem from their predominant distribution in the body in a hypoxic microenvironment. For example, the partial pressure of oxygen in the bone marrow region is typically 1 to 9%, that in adipose tissue is typically 5 to 9%, and that in the umbilical cord is typically 1 to 6% [24]. Because of this, MSCs have evolved a unique ability to adapt to the hypoxic microenvironment, a property that contributes to their extreme resilience under hypoxic conditions. It has been shown that hypoxic induction raised the yield of EVs and enhanced the therapeutic efficacy of EVs significantly compared to normoxic EVs [25,26]. Based on this, we speculate that hy-EVs derived from HUCMSCs may be more suitable for the hypoxic microenvironment of diabetic wounds, further enhancing their biological efficacy. However, there are few studies on the use of hy-EVs on the functions of skin cells and their specific roles and molecular mechanisms during diabetic wound healing.

**Figure 1:**
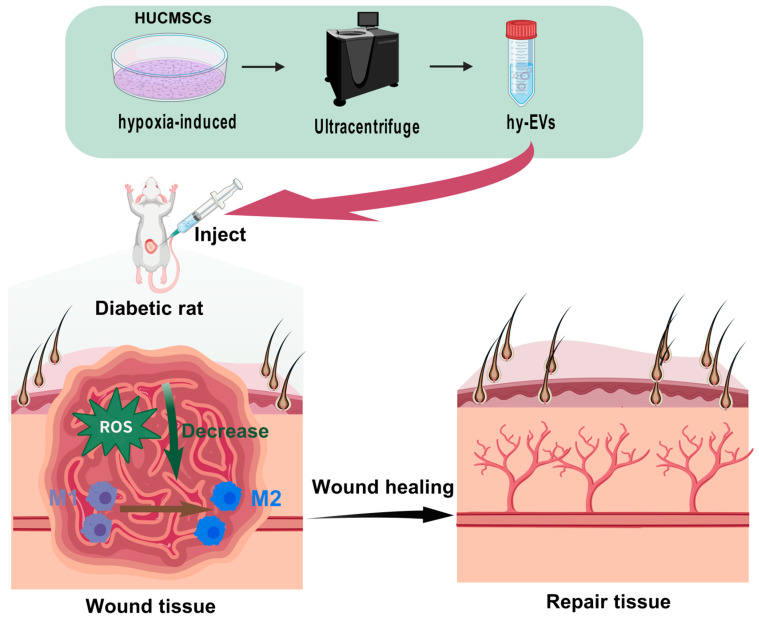
Schematic diagram of the treatment of diabetic wounds with hy-EVs derived from HUCMSCs induced by hypoxia.

The purpose of this study is to explore the functional role and molecular mechanism of hy-EVs in promoting diabetic wound recovery (Figure 1). According to our findings, hy-EVs can facilitate the wound healing by enhancing the functions of HSFs and HUVECs, inducing angiogenesis, regulating macrophage polarization, dampening inflammation, and alleviating oxidative stress. Our findings not only provide new theoretical support for diabetic wound treatment but also lay the foundation for future utilization of EVs in regenerative medicine.

## 2. Method Materials

### 2.1. Cell Culture

The First Affiliated Hospital of Guangxi Medical University granted ethical approval (Approval Number: 2025-E0711, Approval date: 28 March 2025) for the successful acquisition of HUCMSCs. Human umbilical cord samples were collected from full-term healthy donors via cesarean section at The First Affiliated Hospital of Guangxi Medical University, and collection was implemented based on the principles of aseptic technique. The previous study has been taken as reference for extraction, culture and identification of HUCMSCs [12]. HSFs (AW-CH0154, Anweisci, Shanghai, China) and HUVECs (AW-CH0165, Anweisci) were both cultured in high-glucose medium (BL304A, Biosharp, Hefei, China), supplemented with additional glucose to achieve the final glucose concentration of 35 mmol/L. Macrophages were differentiated from human monocyte leukemia cells (AW-CH0359, Anweisci) using phorbol ester (P6741, Solarbio, Beijing, China). M1 macrophages were induced by lipopolysaccharide (L8880, Solarbio) for 48 h. RPMI 1640 medium (BL303A, Biosharp). These cells were maintained in a humidity-controlled incubator containing 5% CO_2_ at 37 °C.

### 2.2. Collection and Characterization of hy-EVs Derived from HUCMSCs

Primary extracted HUCMSCs were used for subsequent experiments after passaging to the 5th generation. When HUCMSCs reached a density of approximately 80%, we changed the medium to a serum-free medium. The n-EVs group was incubated continuously at 37 °C for 48 h under normoxic conditions of 5% carbon dioxide (CO_2_), 21% oxygen (O_2_), and 74% nitrogen (N_2_), while the hy-EVs group was cultured at 37 °C for 48 h with 5% CO_2_, 3% O_2_, and 92% N_2_, and the culture was controlled by a hypoxic incubator (MIC101, COLLRUN), a dual flow meter (DFM3002, COLLRUN), and a modular oxygen monitor (MOM5003, COLLRUN) to precisely control the 3% O_2_ concentration. After 48 h of incubation, the medium was successfully collected and aliquoted into 50 mL centrifuge tubes for density gradient centrifugation. The first centrifugation was performed for 10 min at 300× *g*, then centrifuged for 20 min at 2000× *g* to remove cellular debris. Following this, we passed the supernatant via a 0.22 μm filter to clear nanoscale contaminants. The volume of the supernatant from the initial centrifugation was approximately 200 mL, and the resulting supernatant underwent ultracentrifugation at 4 °C for 70 min at 120,000× *g*, repeated twice. These isolated n-EVs and hy-EVs were meticulously resuspended in 100 µL of Phosphate-Buffered Saline (PBS), these preparations could be used directly in experiments or kept at −80 °C for later applications. Nanoparticle tracking analysis (NTA, (ZetaView PMX 110, Particle Metrix, Meerbusch, Germany)) was employed to comprehensively characterize the distribution of sizes for n-EVs and hy-EVs. Transmission electron microscopy (TEM, (H7650, HITACHI, Tokyo, Japan)) was employed to characterize their microstructure. Western blot analysis was conducted to measure major biomarkers associated with EVs. The examined markers included the positive markers CD63, CD9, CD81, and TSG101, as well as the negative marker Calnexin.

### 2.3. Investigation of hy-EVs Uptake by HUVECs

N-EVs and hy-EVs were labeled with Cell Plasma Membrane Red Fluorescence Staining Kit (PKH26, Beyotime, Shanghai, China). To begin with, the fluorescent dye working solution was used to label the n-EVs and hy-EVs, followed by washing with PBS, centrifugation, and complete removal of unbound dyes. The HUVECs were co-incubated with the fluorescently labeled n-EVs and hy-EVs for 6 h to observe their uptake of these fluorescently labeled n-EVs and hy-EVs. First, tissue samples were fixed for 15 min with 4% paraformaldehyde. After the fixation procedure, we used Actin-Tracker Green-488 (C2201S, Beyotime) for an additional duration of 10 min to visualize the organization of the cytoskeleton. After that, DAPI (C1006, Beyotime) was used to stain the nuclei for 1 min. Following the staining procedures, we used PBS to wash any unbound reagents before imaging. In the end, fluorescence images were taken with a confocal laser scanning microscope (CLSM).

### 2.4. Cell Counting Kit-8 (CCK-8)

To study cellular growth, an equal number of cells were inoculated into each well of a 96-well microplate. N-EVs and hy-EVs were added to the medium at doses of 0, 5, 10, 15, 20, and 25 μg/mL, and cells were incubated. After 24 h of cell incubation, the medium was carefully aspirated and washed gently with PBS. Immediately thereafter, 200 μL of freshly prepared CCK-8 working solution was dispensed into every well. The cells were incubated for 1 h at 37 °C. The density of light absorption in every well was finally detected at 450 nm by a spectrophotometer.

### 2.5. Live–Dead Cell Staining

An equal number of cells were plated into the 48-well plate and separated into three groups: the control (PBS) group, the n-EVs group, and the hy-EVs group. After 24 h, we removed the medium and washed it once with PBS. Then we added the Calcein AM/PI assay working solution (C2015S, Beyotime), followed by incubation for 20 min at 37 °C. We used PBS to wash and then the cells were observed and photo-documented via a microscope.

### 2.6. Transwell Assay

To assess the capacity of cell migration, trypsin was used to digest the cells and subsequently resuspended in serum-free medium. The upper compartment of a 12-well Transwell chamber was seeded with 200 µL of the prepared cell suspension. At the same time, the 800 μL DMEM medium of different components was introduced into the lower chamber for the nutrient supply of the cells. We used a cotton bud to gently discard the cells from the upper chamber after 24 h of incubation. The filter membrane was cleansed using PBS to get rid of any non-adherent cells. Following that, a 10 min fixation was performed on the cells using 4% paraformaldehyde. After a 15 min staining period with crystal violet, the cells were rinsed with PBS. A light microscope was employed to analyze and count the cells in the lower chamber.

### 2.7. Scratch Wound Assay

Wound healing was simulated by culturing cells in 6-well plates in vitro. Once the cells reached full confluence, a straight scratch was made across the monolayer. PBS was used to briefly rinse the cells, and the prepared medium was added according to different treatment groups. Photographs were acquired at spaced intervals of 0 h, 12 h and 24 h to study the wound healing and cell migration. The extent of the wound region was quantitatively measured by the ImageJ software (v1.53t), and the formula ((D0 − Dt)/D0 × 100%) was used. Here, D0 refers to the size of the wound at the beginning, and Dt refers to the wound’s residual size at the set time.

### 2.8. Tube Formation Assay

HUVECs were chosen as the research subjects. First, 100 μL of pre-cooled ABW^®^ Matrigel (0827045, ABW, Shanghai, China) at 4 °C was carefully added to the wells of a pre-chilled 48-well plate. The plate was subsequently left in an incubator to polymerize the Matrigel for 1 h at 37 °C. After that, HUVECs resuspended in the medium were inoculated onto the surface of the polymerized gel, and groups (control, n-EVs, and hy-EVs) were set up with the same number of cells in each group. After incubating the plate for 6 h at 37 °C, the generation of lumen formations on gel was seen and recorded under the optical microscope. ImageJ software was employed to process and analyze all images.

### 2.9. Cell Immunofluorescence

Following the various treatments, the cells were fixed using immunostaining fixative (P0098, Beyotime) for 10 min. Then cells were permeabilized using the immunostaining permeabilizing solution (P0096, Beyotime) for 10 min. Finally, the cells were blocked in immunostaining blocking solution (P0102, Beyotime) for 30 min. Overnight incubation of primary antibodies was performed at 4 °C. The cells were co-incubated with fluorescently labeled secondary antibody (A0516 and A0562, Beyotime) for 1 h. Afterward, DAPI (C1006, Beyotime) was used to stain the cells for 1 min. Finally, the cells were visualized and captured with the microscope. The primary antibodies used were CD86 (26903-1-AP, Proteintech, Wuhan, China), CD206 (18704-1-AP, Proteintech), HIF-1α (BM4083, BOSTER, Wuhan, China), CD31 (11265-1-AP, Proteintech), and VEGFA (19003-1-AP, Proteintech).

### 2.10. ROS Measurement

The 2′,7′-dichlorodihydrofluorescein diacetate fluorescent probe (DCFH-DA, Beyotime) was used to quantify ROS levels in HSFs and HUVECs. A 12-well plate was used to seed the cells, and then the cells were washed gently after 24 h of incubation. Afterwards, ROS staining was carried out. We used CLSM to take fluorescence photos of HSFs and HUVECs, then used ImageJ software to measure and quantify the signals.

### 2.11. Protein Extraction and Western Blot

The cells were harvested from the 6-well plate, with all procedures performed under ice-cold conditions. First, the cells were centrifuged at 4 °C for 10 min at 4000× *g*. The supernatant was carefully removed, and the pellet of cells was retained. The samples were then lysed in RIPA lysis buffer (P0013B, Beyotime) and protein inhibitor (P1005, Beyotime) for 30 min to ensure effective lysis. After being lysed, the sample was centrifuged at 4 °C for 10 min at 12,000× *g*. Following the separation by centrifugation, the supernatant was harvested. The obtained supernatant was mixed with SDS-PAGE loading buffer (P0015, Beyotime) at a predetermined ratio and heated for 10 min at 100 °C. The samples were stored at −20 °C for subsequent experiments. Subsequently, SDS-PAGE electrophoresis buffer (P0014D, Beyotime) was used for electrophoresis. Then, the membranes (ISEQ00010, Merck Millipore, Billerica, MA, USA) were employed for protein transfer for 20 min, followed by blocking them with blocking buffer (P0023B, Beyotime) for another 20 min. After overnight incubation of primary antibodies at 4 °C, secondary antibody (A0208, Beyotime) was applied for 1 h. The target protein band was detected using the ECL reagent kit (P0018M, Beyotime), and the gray value was measured using ImageJ to represent the protein expression level. The internal reference was used as the loading control to normalize the data. The primary antibodies included HIF-1α (BM4083, BOSTER), CD31 (11265-1-AP, Proteintech), VEGFA (19003-1-AP, Proteintech), and Alpha Tubulin (14555-1-AP, Proteintech).

### 2.12. Animal Experiments

All animal-related protocols were executed following the specified guidelines established by the Animal Experiment Center of Guangxi Medical University (Approval Number: 202505004, Approval date: 17 June 2025). The experimental models consisted of Sprague-Dawley (SD) male rats, eight-week-old SD rats were housed under standard laboratory animal conditions and were acclimatized to the new environment for 1 week. Following an overnight fasting period, all rats received an intraperitoneal administration of streptozotocin (S8050, Solarbio) at 50 mg/kg to induce diabetes. Blood glucose level was measured daily until stabilized after one week. All animals with serum glucose concentrations more than 16.67 mmol/L were used in the study. A diabetic skin defect model with a diameter of approximately 10 mm was prepared on the back of rats following intraperitoneal anesthesia using 2% pentobarbital (P3761, Sigma-Aldrich, St. Louis, MO, USA) at a dose of 200 μL/kg. The rats were randomly split into three groups, which were the negative control group (100 μL PBS by subcutaneous injection, *n* = 6), the n-EVs group (100 μL n-EVs at 20 μg/mL by subcutaneous injection, *n* = 6), and the hy-EVs group (100 μL hy-EVs at 20 μg/mL by subcutaneous injection, *n* = 6). A digital camera monitored the advancement of wound healing at days 0, 3, 6, 9 and 12 post-injury. The wound area was quantitatively estimated at each time point using ImageJ software. Euthanasia using overdose anesthesia after completion of experiments.

### 2.13. Histological Analysis

On the 12th day post-treatment, the wound tissues from rat skin were collected and fixed. After 48 h of fixation, they were dehydrated, embedded in paraffin, and sectioned for future analysis. Afterwards, hematoxylin–eosin (HE) staining was performed for general histopathological features. We performed Masson’s trichrome staining to quantify the amount of collagen present in the wound tissues.

### 2.14. Immunohistochemical Analysis and Immunofluorescence Analysis

Sections were dewaxed, hydrated, antigenically repaired, and blocked for immunohistochemical staining. After an overnight exposure to the primary antibodies, the sections were subsequently exposed to the secondary antibodies for one hour, and counterstained with DAPI, visualized and imaged with a microscope. For immunofluorescence staining, fluorescently labeled secondary antibodies were utilized during the incubation step. All other procedures were similar to those used for immunohistochemistry staining except that the samples were protected from light throughout the process. The primary antibodies included CD31 (11265-1-AP, Proteintech), VEGFA (19003-1-AP, Proteintech), TNF-α (17590-1-AP, Proteintech), CD86 (26903-1-AP, Proteintech), and CD206 (18704-1-AP, Proteintech).

### 2.15. Statistical Analysis

We used GraphPad Prism 8.0 software for all statistical analysis, and the data in this study were repeated at least three times independently and expressed as mean ± standard deviation (SD). The independent Student’s *t*-test was used to compare the two groups. For three or more groups, one-way analysis of variance (ANOVA) was applied. A *p*-value < 0.05 was used to define statistical significance in all evaluations, indicating a significant difference between groups.

## 3. Results

### 3.1. Isolation and Identification of hy-EVs Derived from HUMSCs

**Figure 2:**
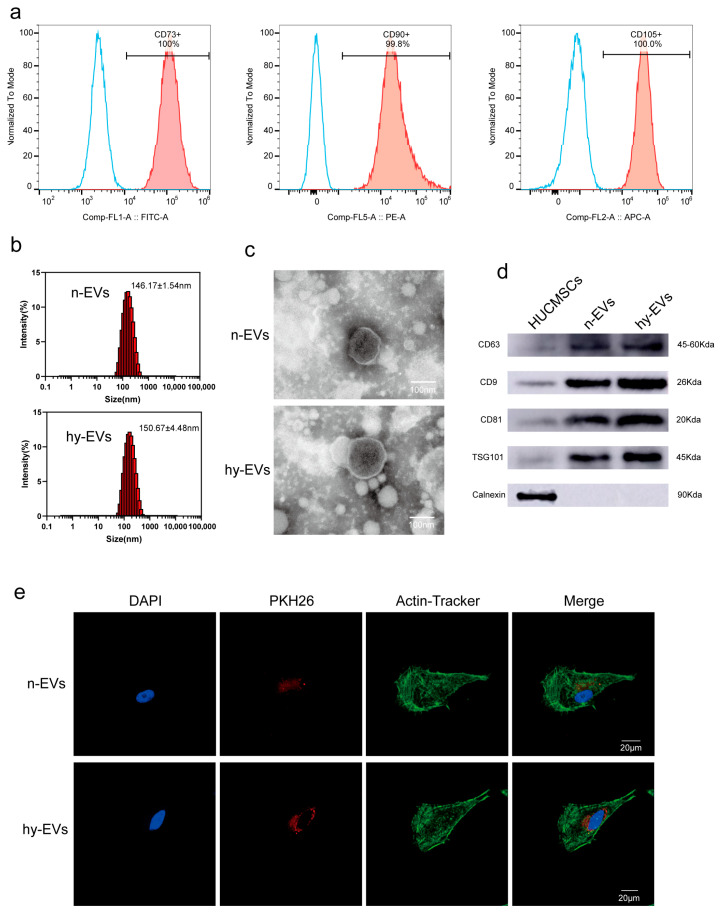
Extraction and identification of HUCMSCs and their EVs. (**a**) Identification of HUCMSCs markers CD73, CD90, and CD105 by flow cytometry. (**b**) NTA measurement of n-EVs and hy-EVs. (**c**) Morphological features and sizes of n-EVs and hy-EVs were analyzed under TEM. (**d**) Western blot analysis of surface protein markers CD63, CD9, CD81, TSG101and negative marker Calnexin of EVs (original Western blot images can be found in Figure S4). (**e**) CLSM images of HUVECs (green) co-incubated with n-EVs (red) and hy-EVs (red) for 6 h, Nuclei were stained with DAPI (blue).

We took HUCMSCs from human umbilical cord tissue to grow them in culture. Flow cytometric analysis was used for the assessment of expression of specific surface markers of HUCMSCs. Flow cytometric analysis demonstrated that the expression rates of surface markers in HUCMSCs were 100% for CD73+, 99.8% for CD90+, and 100% for CD105+ (Figure 2a). In addition, we analyzed the expression levels of surface markers associated with monocytes, B lymphocytes, hematopoietic stem cells, and other hematopoietic cells. The results showed that the expression of CD11b+, CD19+, CD34+, CD45+ and HLA-DR+ was negligible, with positive rates of 0.066%, 0.43%, 0.29%, 0.047% and 0.41% (Supplementary Figure S1), which met the identification requirements for HUCMSCs. These results showed that HUCMSCs were successfully extracted from umbilical cord tissue. Thereafter, we induced HUCMSCs by normoxic and hypoxic conditions, respectively, and we found that there was no significant difference in morphology between the two cells after 48 h of culture (Supplementary Figure S2). After that, we used ultracentrifugation to extract and purify n-EVs and hy-EVs from HUCMSCs. NTA showed that the average particle sizes of n-EVs and hy-EVs were approximately 146.17 ± 1.54 nm and 150.67 ± 4.48 nm, respectively, which confirmed that both n-EVs and hy-EVs were within the expected size range (Figure 2b). Through TEM observation, the n-EVs and hy-EVs exhibited a unique cup-shaped structure and possessed an intact vesicle membrane (Figure 2c). Furthermore, Western blot analysis verified the high expression of the major markers CD63, CD9, and TSG101 in the purified n-EVs and hy-EVs, while Calnexin was negatively expressed (Figure 2d). Overall, the analysis revealed that the n-EVs and hy-EVs were successfully extracted. To verify whether HUVECs can take up n-EVs and hy-EVs, we used PKH26 to label n-EVs and hy-EVs, then co-incubated with HUVECs. After co-incubation for 6 h, the results of immunofluorescence showed that both n-EVs and hy-EVs were successfully taken up by HUVECs. HUVECs that took up hy-EVs exhibited stronger fluorescence signals, which surrounded the nucleus (Figure 2e). The findings suggested that HUVECs were able to take up hy-EVs more easily, which might potentially enhance their functional efficacy in biological processes.

### 3.2. hy-EVs Derived from HUMSCs Enhanced the Functions of HSFs

**Figure 3:**
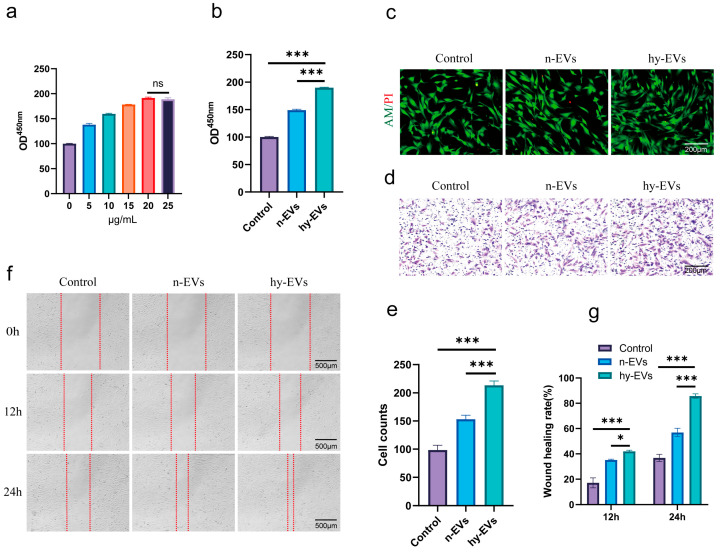
Hy-EVs enhanced the proliferation and migration of HSFs. (**a**) Analysis of CCK-8 results after HSFs were treated with different concentrations of hy-EVs for 24 h (*n* = 3 per group). (**b**) Analysis of CCK-8 results of HSFs after different treatments in each group for 24 h (*n* = 3 per group). (**c**) Live–dead cell staining of HSFs after different treatments in each group for 24 h (*n* = 3 per group). (**d**) Transwell assay to analyze the migration of HSFs among different groups after different treatments for 24 h and (**e**) quantitative analysis of cell migration (*n* = 3 per group). (**f**) Scratch assay to analyze the healing of HSFs among different groups after different treatments for 12 h and 24 h and (**g**) quantitative analysis of the healing rate of the scratch assay (*n* = 3 per group). Data are mean ± SD, ns: not significant, * *p* < 0.05, *** *p* < 0.001. All *p* values were obtained by one-way ANOVA.

The wound healing process involves several cell types including HSFs that are critical functional cells in this process [27]. First, we investigated the impact of hy-EVs on HSFs’ functions. We used the CCK-8 assay to assess the role of hy-EVs on the proliferation of HSFs. The data revealed that hy-EVs promoted the proliferation of HSFs in a dose-dependent manner under high-glucose conditions (Figure 3a). Specifically, the proliferative effect of hy-EVs increased with concentration in the range of 0–20 μg/mL and plateaued at the concentration of 20 μg/mL, and the proliferative effect did not increase significantly with increasing concentration of hy-EVs. Thus, we concluded that 20 μg/mL was the optimal concentration for further study. Meanwhile, we compared the effect of the Control group and n-EVs group on the proliferation of HSFs at the same concentration. We found that hy-EVs had a stronger proliferative effect on HSFs at the same concentration (Figure 3b). Live–dead cell staining indicated that hy-EVs had good biocompatibility and verified their proliferative effect on HSFs (Figure 3c). We performed a Transwell assay to assess the migration ability. The study revealed that hy-EVs considerably enhanced the migration ability of HSFs compared to the control and n-EVs groups at 24 and 48 h (Figure 3d,e). Additionally, the scratch assay results indicated that the rate of wound healing in the control and n-EVs groups was markedly lower than the hy-EVs group at 24 and 48 h, indicating that hy-EVs could greatly promote the migration of HSFs (Figure 3f,g). Our findings demonstrated that hy-EVs were essential for enhancing the function of HSFs, which may aid in enhancing diabetic wound healing.

### 3.3. hy-EVs Derived from HUMSCs Enhanced the Functions of HUVECs

**Figure 4:**
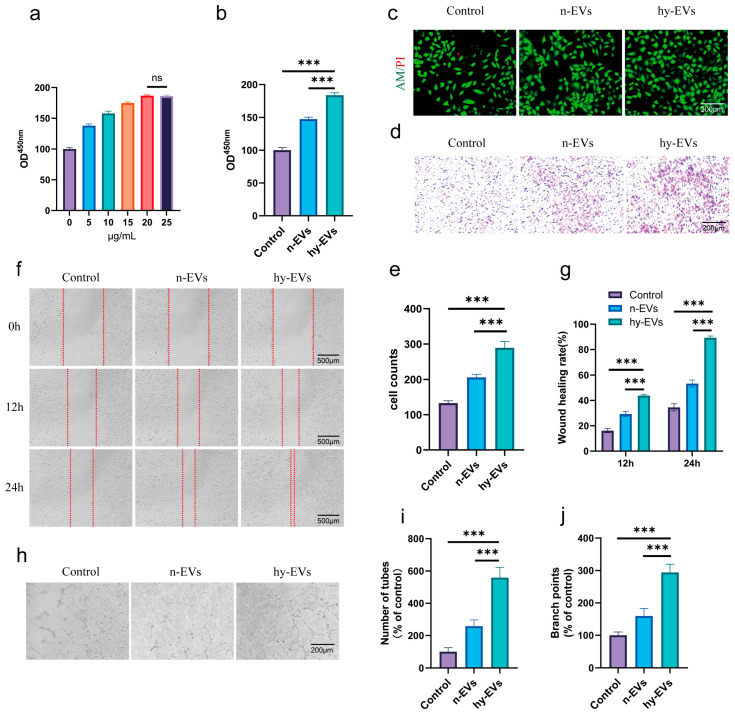
Hy-EVs enhanced the proliferation, migration, and tube formation of HUVECs. (**a**) Analysis of CCK-8 results of HUVECs treated with different concentrations of hy-EVs for 24 h (*n* = 3 per group). (**b**) Analysis of CCK-8 results of HUVECs in each group after different treatments for 24 h (*n* = 3 per group). (**c**) Live–dead cell staining of HUVECs in each group after different treatments for 24 h (*n* = 3 per group). (**d**) Transwell assay to analyze the migration of HUVECs among groups after different treatments for 24 h and (**e**) quantitative analysis of cell migration (*n* = 3 per group). (**f**) Scratch assay to analyze the wound healing of HUVECs among groups after different treatments for 12 h and 24 h and (**g**) quantitative analysis of the wound healing rate in the scratch assay (*n* = 3 per group). (**h**) In vitro tube formation assay to analyze the tube-forming ability of vascular HUVECs in each group after different treatments and quantitative analysis of (**i**) number of tubes and (**j**) branch points (*n* = 3 per group). Data are mean ± SD, ns: not significant, *** *p* < 0.001. All *p* values were obtained by one-way ANOVA.

It is widely recognized that angiogenesis serves as a pivotal factor in diabetic wound healing, and promoting angiogenesis continues to be a significant challenge in diabetic wounds [28]. Therefore, we tested the impact of hy-EVs on the capabilities of HUVECs. Similarly, we used the CCK-8 assay to assess the impact of hy-EVs on proliferation of HUVECs. Consistent with the results of the study on HSFs, hy-EVs markedly enhanced the proliferation of HUVECs in a dose-dependent manner (Figure 4a). The optimal concentration of hy-EVs for promoting the proliferation of HUVECs was 20 μg/mL. At the same concentration, hy-EVs exhibited stronger proliferative effects on HUVECs contrasted with both the control group and the n-EVs group (Figure 4b). Live–dead cell staining further corroborated the good biocompatibility of hy-EVs and the proliferative effect on HUVECs (Figure 4c). The Transwell assay validated the proliferative impact of hy-EVs on the migration of HUVECs (Figure 4d,e). Additionally, we used the scratch wound healing experiment to assess the effect of hy-EVs on HUVECs. Similarly to the findings of the study on HSFs, the scratch healing rate of HUVECs was lowest in the control group, followed by the n-EVs group, while the scratch healing rate was significantly increased by hy-EVs treatment (Figure 4f,g). Given the positive correlation between angiogenesis and wound healing, we further investigated whether hy-EVs could promote angiogenesis in vitro. The results of our study showed that the highest number of lumens formed in the hy-EVs group, although n-EVs treatment increased lumen formation, it was markedly lower than the hy-EVs group, while the control group exhibited the fewest lumens (Figure 4h–j). The results confirmed the ability of hy-EVs to stimulate angiogenesis in vitro. Collectively, these findings revealed that hy-EVs had significant characteristics of promoting the proliferation, migration, and angiogenesis of HUVECs, thereby contributing to the promotion of wound healing in diabetes.

### 3.4. hy-EVs Exhibited Anti-Inflammatory Effect and Mediate Angiogenesis Through the HIF-1α Pathway

**Figure 5:**
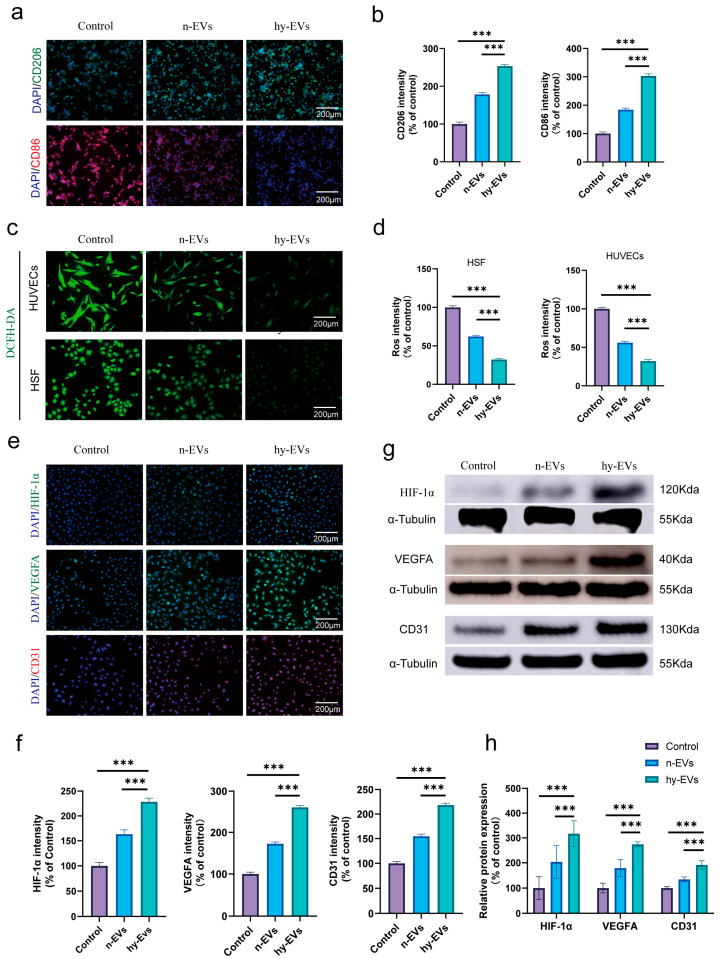
Anti-inflammatory and pro-angiogenic effects of hy-EVs in vitro. (**a**) Immunofluorescent staining of CD86 (red) and CD206 (green) and (**b**) quantitative analysis of their fluorescence intensities (*n*  =  3 per group). (**c**) ROS fluorescent staining of HSFs and HUVECs in different treatment groups and (**d**) quantitative analysis of their fluorescence intensities (*n*  =  3 per group). (**e**) Immunofluorescence staining of HIF-1α (green), VEGFA (green) and CD31 (red) expression in different treatment groups, and the nuclei were stained with DAPI (blue); (**f**) quantitative analysis of their fluorescence intensities (*n* = 3 per group). (**g**) Western blot analysis of the expression levels of HIF-1α, VEGFA and CD31 in different treatment groups (original Western blot images can be found in Figure S4), and (**h**) their quantitative analysis (*n*  =  3 per group). Data are mean ± SD, *** *p* < 0.001. All *p* values were obtained by one-way ANOVA.

Recent studies have shown that immune imbalance in diabetic wounds constitutes a critical factor influencing the recovery progression of such wounds [29]. Based on this, we further explored the impact of hy-EVs on macrophages. The results showed that the expression of CD86 (a marker for M1 macrophages) was markedly reduced after hy-EVs treatment, while the expression of CD206 (a marker for M2 macrophages) was notably increased, indicating that hy-EVs can modulate the transition of macrophages from M1 (pro-inflammatory) to M2 (anti-inflammatory) phenotype (Figure 5a,b), thereby regulating the immunological microenvironment associated with diabetic wounds.

Recent research has pointed out that excessive ROS accumulation will significantly impair the regular wound recovery process [30]. Therefore, we further evaluated whether hy-EVs can lessen the ROS levels. The outcomes revealed that the fluorescence intensity of ROS was highest in the Control group and that hy-EVs treatment could effectively inhibit the production of ROS. In contrast, n-EVs treatment showed a relatively limited ability to scavenge ROS (Figure 5c,d). This indicated that hy-EVs can effectively scavenge ROS, which may be the key mechanism for relieving excessive oxidative stress in diabetic wounds.

In addition, to verify whether hypoxia activates HIF-1α, we first evaluated the effect of hypoxia induction on HIF-1α expression in HUCMSCs, and we found that HIF-1α was highly expressed in hypoxia-induced HUCMSCs after 48 h, whereas normoxic cultured HUCMSCs were low in HIF-1α expression (Figure S3). Our previous experimental results have confirmed the in vitro angiogenic properties of hy-EVs. Therefore, we evaluated whether hy-EVs promote angiogenesis by activating HIF-1α and assessed the effects of different treatments on the expression levels of VEGFA and CD31. Immunofluorescence results showed that the expression levels of HIF-1α, VEGFA and CD31 were significantly up-regulated after treatment with hy-EVs (Figure 5e,f). Consistent with the results of immunofluorescence experiments, protein blotting experiments further confirmed that hy-EVs significantly increased the expression levels of HIF-1α, VEGFA and CD31 (Figure 5g,h). This indicated that hy-EVs promoted angiogenesis by activating the HIF-1α pathway.

In summary, our study demonstrated that hy-EVs can play multiple roles, such as inducing a phenotypic shift from M1-type to M2-type in macrophages, attenuating oxidative stress, exerting anti-inflammatory effects, and stimulating angiogenesis. All these factors were crucial in facilitating diabetic wound healing.

### 3.5. hy-EVs Promoted the Healing of Diabetic Wounds In Vivo

**Figure 6:**
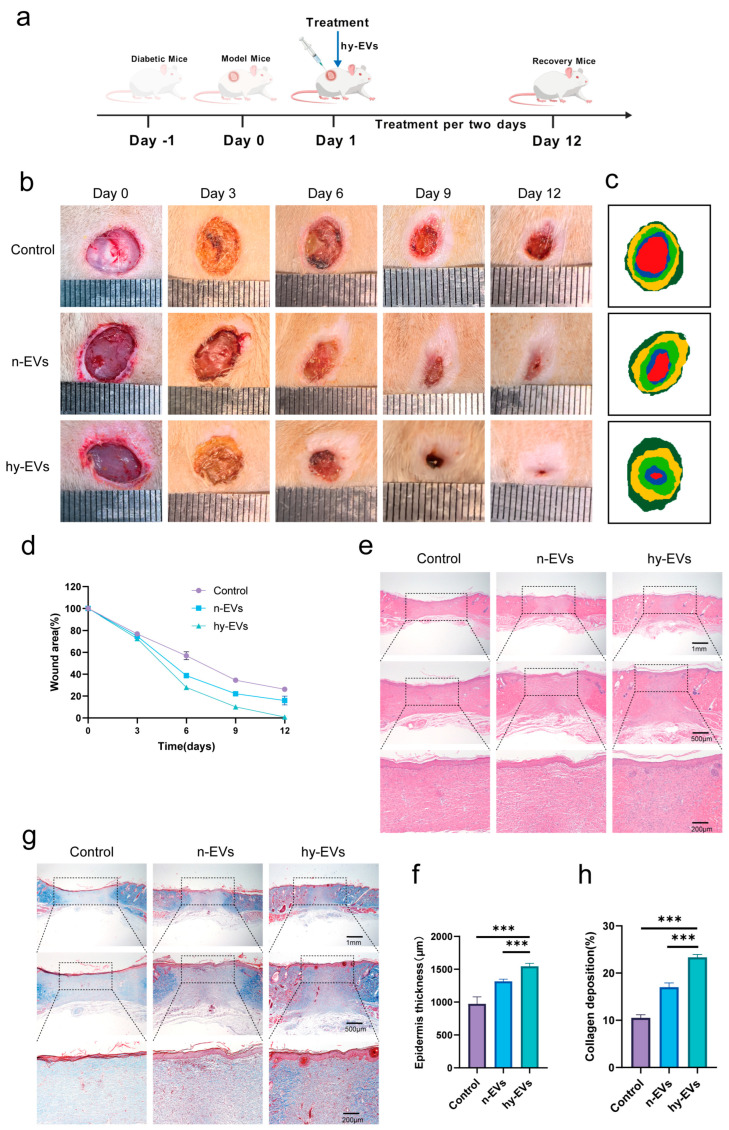
Hy-EVs accelerated the healing of diabetic wounds in vivo. (**a**) Establishment of a skin defect model in diabetic rats and subsequent treatment regimens. (**b**) Representative images of the wounds on days 0, 3, 6, 9, and 12. (**c**) Simulated images of the wound healing process. (**d**) Quantitative analysis of the wound healing rate in each group (*n*  =  6 per group). (**e**) HE staining of the wound tissue in each group on day 12 and (**f**) quantitative analysis of its epidermis thickness (*n*  =  6 per group). (**g**) Masson staining of the wound tissue in each group on day 12 and (**h**) quantitative analysis of its collagen deposition (*n*  =  6 per group). Data are mean ± SD, *** *p* < 0.001. All *p* values were obtained by one-way ANOVA.

**Figure 7:**
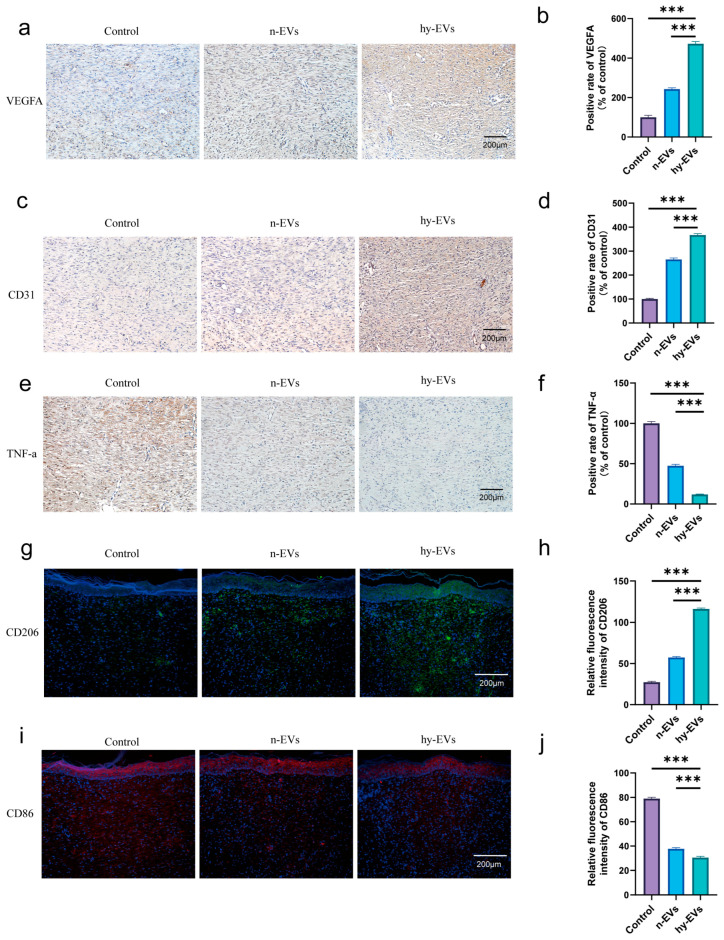
Immunohistochemical and immunofluorescent analyses of wound tissues. (**a**) Immunohistochemical analysis of CD31 and (**b**) quantitative analysis (*n* = 6 per group). (**c**) Immunohistochemical analysis of VEGFA and (**d**) quantitative analysis (*n* = 6 per group). (**e**) Immunohistochemical analysis of TNF-α and (**f**) quantitative analysis (*n* = 6 per group). (**g**) Immunofluorescent analysis of CD206 and (**h**) quantitative analysis (*n* = 6 per group). (**i**) Immunofluorescent analysis of CD86 and (**j**) quantitative analysis (*n* = 6 per group). Data are mean ± SD. *** *p* < 0.001. All *p* values were obtained by one-way ANOVA.

Modeling the diabetic rats was performed so that we could investigate the function of hy-EVs during wound healing. Following this, a cutaneous wound about 10 mm in diameter was inflicted on the rats’ dorsal area, and a subcutaneous injection of 100 μL of 20 μg/mL hy-EVs was performed on the wound edge every two days (Figure 6a). Wound healing was investigated with the aid of macroscopic images taken on days 0, 3, 6, 9 and 12 post-modeling. Throughout the experiment, it was observed that the hy-EVs group had the fastest rate of wound healing, while the Control group was the slowest (Figure 6b–d). Notably, treatment with n-EVs could indeed significantly promote the regenerative process of wounds in diabetes. However, compared with the hy-EVs group, the healing process was significantly delayed, which fully indicated that hy-EVs played an important role in facilitating diabetic wound healing. Histological analysis by HE staining also confirmed these findings (Figure 6e,f). On day 12, the wounds in the hy-EVs group were nearly fully healed, and new hair follicles were visible. In contrast, the control group and the n-EVs group both showed relatively limited skin thickness and hair follicle regeneration. The findings from Masson’s trichrome staining demonstrated that the hy-EVs group exhibited the most abundant collagen deposition (Figure 6g,h), indicating that hy-EVs can promote collagen deposition and consequently enhance diabetic wound healing. Additionally, we measured the expression levels of angiogenesis-associated factors like VEGFA and CD31. Immunohistochemical staining of excised tissues indicated that hy-EVs notably upregulated the expression levels of VEGFA (Figure 7a,b) and CD31 (Figure 7c,d) in vivo to promote angiogenesis in diabetic wounds. Meanwhile, we also detected the expression of the inflammatory factor TNF-α (Figure 7e,f), which was markedly decreased in the hy-EVs group, indicating that hy-EVs significantly reduced inflammation in the wound. In the hy-EVs group, we found that CD206 expression was highest (Figure 7g,h), while CD86 expression was lowest (Figure 7i,j). This suggested that hy-EVs can be used to modulate the immune response and facilitate wound healing by modulating the shift from M1 to M2 macrophage polarization. Collectively, these findings emphasized the important role of hy-EVs in enhancing diabetic wound healing.

## 4. Discussion

Diabetes wounds are still a major clinical treatment challenge with high incidence and complex etiology. They occur as a result of hyperglycemia, vascular complications, persistent inflammation, and infection susceptibility [31,32]. Standard treatments such as glycemic control and wound debridement usually lack efficacy and thus have a high rate of recurrence and severe treatment difficulty [31,33]. Patients suffering from diabetic wounds experience severe physical pain and psychological distress. Thus, better treatment options are warranted [34]. In this study, hy-EVs derived from HUMSCs effectively promoted diabetic wound repair.

Diabetic wounds are subject to non-enzymatic glycosylation of proteins and lipids with glucose due to their high-glucose microenvironment, resulting in the formation of glycosylation end-products (AGEs) [35,36]. AGEs impair cell functions, inhibit the proliferation and migration capability of HSFs, and reduce collagen synthesis, while promoting inflammatory responses and exacerbating vascular lesions, leading to delayed wound healing [37,38]. Our in vitro findings suggested that the functions of HSFs were impaired in a high-glycemic environment, which delayed scratch healing. Notably, while EVs produced under normoxic conditions enhanced the functions of HSFs, they were less effective than those produced under hypoxic conditions. On the contrary, treatment with hy-EVs significantly improved the functions of HSFs, their proliferation and migration capacities that were essential for wound healing. In vivo study suggested that treatment with hy-EVs significantly promoted the growth of damaged skin, facilitated hair follicle restoration and collagen accumulation, and ultimately accelerated the diabetic wound healing.

One of the core issues linked to diabetic wounds is the impairment of angiogenesis, the presence of long-term hyperglycemia damages HUVECs and triggers vasculopathy, leading to ischemia, hypoxia, and an insufficient supply of nutrients to the local tissues, thus severely affecting the healing process of the wounds [39,40]. Therefore, promoting angiogenesis is a critical therapeutic strategy for diabetic wounds. MSCs have been shown to promote angiogenesis through a mechanism enhanced by hypoxia [41]. Hypoxia preconditioning stabilizes HIF-1α expression in MSCs, and the stabilized HIF-1α binds to the downstream target gene VEGFA and directly upregulates its transcriptional level [42,43]. hy-EVs can enhance HIF-1α activity in HUVECs by carrying specific miRNAs. For example, miR-612 was significantly upregulated in hypoxia-induced olfactory mucosa MSCs-derived EVs. miR-612 binds to the 3′UTR region of TP53 mRNA in target cells and inhibits its expression, which in turn regulates endothelial cell angiogenesis by promoting HIF-1α and VEGF expression [44]. In a recent study, miR-210-3p was found to be up-regulated in exosomes of hypoxia-induced MSCs through HIF-1α, while miR-210-3p could hinder EFNA3 expression, thereby activating the PI3K/AKT pathway and significantly enhancing proliferation, migration and angiogenesis of HUVECs [45]. In another study, hypoxia-preconditioned MSCs promoted angiogenesis through activation of the Ras/Erk pathway by activating HIF-1α-mediated enhancement of exosome miR-126 production and inhibition of Sprouty-related EVH1 structural domain-containing protein 1 (SPRED1) activity [46]. These studies suggest that HIF-1α activation is critical for hypoxia induction, and a similar pathway may exist in hy-EVs. Our study confirmed that the expression of HIF-1α was significantly upregulated in hypoxia-induced HUCMSCs, whereas HIF-1α was lowly expressed in normoxia-induced HUCMSCs. Meanwhile, hy-EVs significantly enhanced the functional properties of HUVECs, especially in proliferation, migration and angiogenesis. hy-EVs significantly upregulated the expression level of HIF-1α in HUVECs, and promoted the expression of the downstream target gene, VEGFA, whereas n-EVs, which lacked the core transcription factor, HIF-1α, had a weaker effect on promoting angiogenesis. The n-EVs, lacking the core transcription factor HIF-1α, had a weaker angiogenic effect. This result was further supported by a recent study in which myocardial capillary density was elevated in the hypoxia-treated group compared with the normoxia group in a porcine model of myocardial ischemia and was accompanied by sustained activation of the Akt/ERK pathway [47]. Sequencing of miRNAs from exosomes derived from BMSCs treated with hypoxia and normoxia revealed considerable differences in the composition of exosomal miRNAs, suggesting that miRNAs are selectively packaged into exosomes after 48 h of hypoxia induction and that upregulated miRNAs are mainly associated with proliferation and angiogenesis [48]. Both our in vitro and in vivo studies demonstrated that hy-EVs promote the expression of VEGFA and CD31 by activating the HIF-1α pathway. Collectively, these findings suggest that hy-EVs promote diabetic wound healing by enhancing angiogenesis.

According to the existing investigations, M1 macrophage overactivation and their consistent inflammatory response on diabetic wounds are among the major contributors to delayed wound healing [49,50]. An emerging therapeutic strategy is to facilitate wound healing by modulating the macrophage polarization from M1 to M2, diminishing the discharge of inflammatory mediators like TNF-α, and thereby remodeling the wound microenvironment [51,52]. Hy-EVs are enriched in specific functional miRNAs than n-EVs, which play a key role in regulating macrophage polarization. For example, miR-21 exosome delivery in hypoxia-preconditioned adipose-derived mesenchymal stem cells (ASCs) contributes to M2-like polarization, miR-21 delivery-induced M2 polarization may be mediated by the PI3K/Akt pathway [53]. In a study of inflammatory or infectious osteolysis, miR-210-3p from EVs produced by hypoxia-induced dental pulp stem cells (DPSCs) impeded both osteoclastogenesis and macrophage inflammatory responses by inhibiting NF-kB1 expression [54]. In another study, miR-216a-5p was upregulated in hy-EVs, and exosomal miR-216a-5p promoted macrophage M2-like polarization by regulating HMGB1 and modulating the HMGB1/TLR4/NF-κB axis [55]. In order to achieve sustained release of exosomes, some researchers achieved regulation of macrophage conversion to the M2 phenotype by hydrogel loading of exosomes produced by hypoxia-induced BMSCs, this effect was achieved because miR-4645-5p in the exosomes and the antioxidant properties of the hydrogel itself combined to inhibit SREBP2 activity in macrophages [56]. These studies suggest that hy-EVs can modulate macrophage polarization by delivering miRNAs that block pro-inflammatory pathways or activate anti-inflammatory pathways. Our in vitro studies demonstrated that hy-EVs effectively regulated macrophage polarization by converting inflammation-induced M1 macrophages into M2-polarized macrophages with inflammatory suppression and tissue regeneration capacities, and the results of in vivo studies similarly demonstrated this. These findings showed that hy-EVs can significantly reduce local inflammation and accelerate diabetic wound healing by regulating the immune response of macrophages. Moreover, in the hyperglycemic environment, the elevated oxidative stress is an important characteristic of diabetic wound microenvironment, and the excessive generation of ROS and imbalance of the antioxidant system further aggravate the wound injury, which is one of the core mechanisms for triggering persistent inflammatory response [57,58]. Therefore, effective clearance of excessive ROS and enhancement of the cellular resistance to oxidative stress may be a promising therapeutic strategy. EVs can directly reduce ROS levels by transferring miRNAs and antioxidant enzyme mRNAs or proteins that activate antioxidant pathways in recipient cells [59]. For example, MSCs-EVs are enriched with antioxidant miRNAs and exhibit significant antioxidant activity, as evidenced by an increase in the activity of antioxidant enzymes, such as superoxide dismutase (SOD), while the Nrf2 signaling pathway is involved in the antioxidant effects of EVs treatment [60]. A study found that hy-EVs could inhibit the PI3K/AKT/TOR signaling pathway via miR214-3p, promote the restoration of mitochondrial membrane potential and ATP, and inhibit the production of mitochondrial reactive oxygen species [61]. These studies suggest that n-EVs are enriched with antioxidant miRNAs, and hy-EVs are more therapeutically effective in antioxidant treatment due to the specificity of hypoxia-induced miRNAs production. This was further confirmed by our study showing that hy-EVs significantly enhanced cellular antioxidant capacity while reducing ROS production. This results in an improved local wound microenvironment and an optimal environment for tissue regeneration, demonstrating the essential role of microenvironmental regulation in tissue repair.

In summary, this study validated the effectiveness of hy-EVs on diabetic wound healing. Our findings suggest that hy-EVs enhanced the function of HSFs and HUVECs. Meanwhile, angiogenesis was significantly promoted, and the HIF-1α pathway may play a key role. In addition, modulation of M1 macrophage to M2 polarization immunomodulation and attenuation of oxidative stress were achieved, and these effects may be achieved by delivering specific miRNAs and proteins, among others. These multifaceted therapeutic effects highlight the great potential value of hy-EVs in the treatment of diabetic wounds. However, there are some limitations of our study. First, our study mainly focused on angiogenesis, immunomodulation, and improvement of wound microenvironment in the wound microenvironment, and despite the importance of these directions, we did not perform sequencing analysis of miRNAs, and the molecular mechanisms of their effects still need to be explored in depth. Secondly, the wound pathology characteristics of diabetic rat models differ somewhat from those of human diabetic chronic wounds, which may affect the translation of research results to clinical practice. In the future, we will endeavor to overcome these shortcomings and further improve the experimental design and mechanism exploration, aiming to provide a more comprehensive and reliable scientific basis for the treatment of diabetic wounds.

## 5. Conclusions

In this study, we successfully isolated HUCMSCs and induced the production of EVs under hypoxic conditions, characterized these vesicles and evaluated their curative potential in diabetic wound repair. Our research demonstrates that hy-EVs markedly promote diabetic wound healing. Specifically, hy-EVs promote angiogenesis by activating the HIF-1α pathway. In addition, regulating macrophage polarization from M1-type to M2-type, attenuating inflammation, and alleviating oxidative stress comprehensively improved the diabetic wound microenvironment, and these effects may be achieved by delivering specific miRNAs and proteins, among others. These findings suggest that hy-EVs have great potential as an innovative therapeutic tool to address impaired healing of diabetic wounds.

## Data Availability

The original contributions presented in this study are included in the article/Supplementary Material. Further inquiries can be directed to the corresponding author(s).

## Supplementary Materials

The following supporting information can be downloaded at: https://www.mdpi.com/article/10.3390/biom15111504/s1, Figure S1: Flow cytometric analysis assessed the expression levels of surface markers associated with monocytes, B lymphocytes, hematopoietic stem cells, and other hematopoietic cells; Figure S2: Morphological changes in cells of normoxic cultured and hypoxia-induced HUCMSCs after 48 h in light microscopy; Figure S3: Changes in HIF-1A expression after 48 h in normoxic cultured HUCMSCs and hypoxia-induced HUCMSCs; Figure S4: Original Western blot images for Figure 2d and Figure 5g.
