# Supplementary material for: Hypoxia-Induced Extracellular Vesicles Derived from Human Umbilical Cord Mesenchymal Stem Cells Regulate Macrophage Polarization and Enhance Angiogenesis to Promote Diabetic Wound Healing
Source: Biomolecules. 2025 Oct 24;15(11):1504. doi: 10.3390/biom15111504 (PMC12650418; doi:10.3390/biom15111504)
Supplement: Supplementary file 1 [file biomolecules-15-01504-s001.zip › biomolecules-3913761-supplementary.pdf]

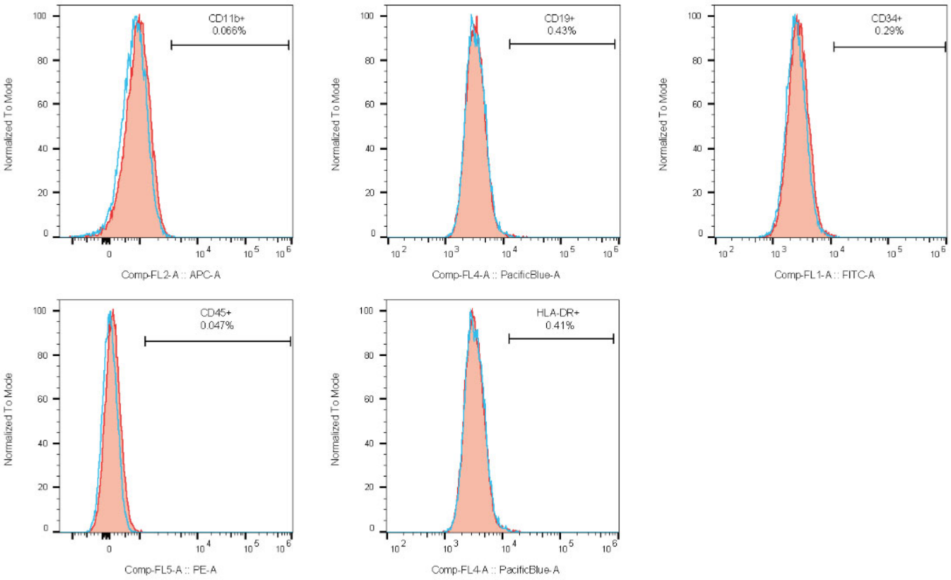

Figure S1. Flow cytometric analysis assessed the expression levels of surface markers associated with monocytes, B lymphocytes, hematopoietic stem cells, and other hematopoietic cells.

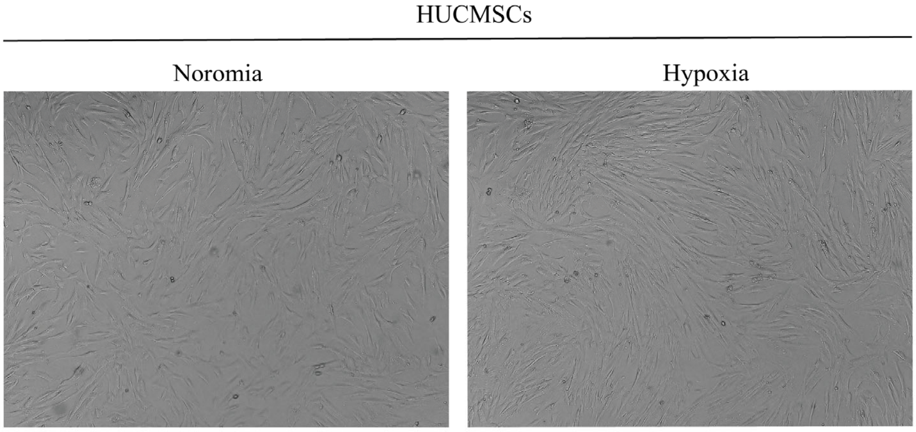

Figure S2. Morphological changes in cells of normoxic cultured and hypoxia-induced HUCMSCs after 48 hours in light microscopy.

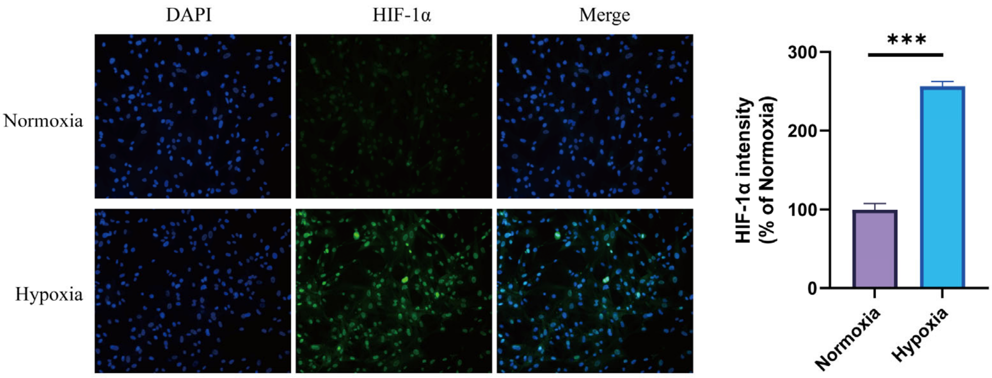

Figure S3. Changes in HIF-1A expression after 48 hours in normoxic cultured HUCMSCs and hypoxia-induced HUCMSCs.

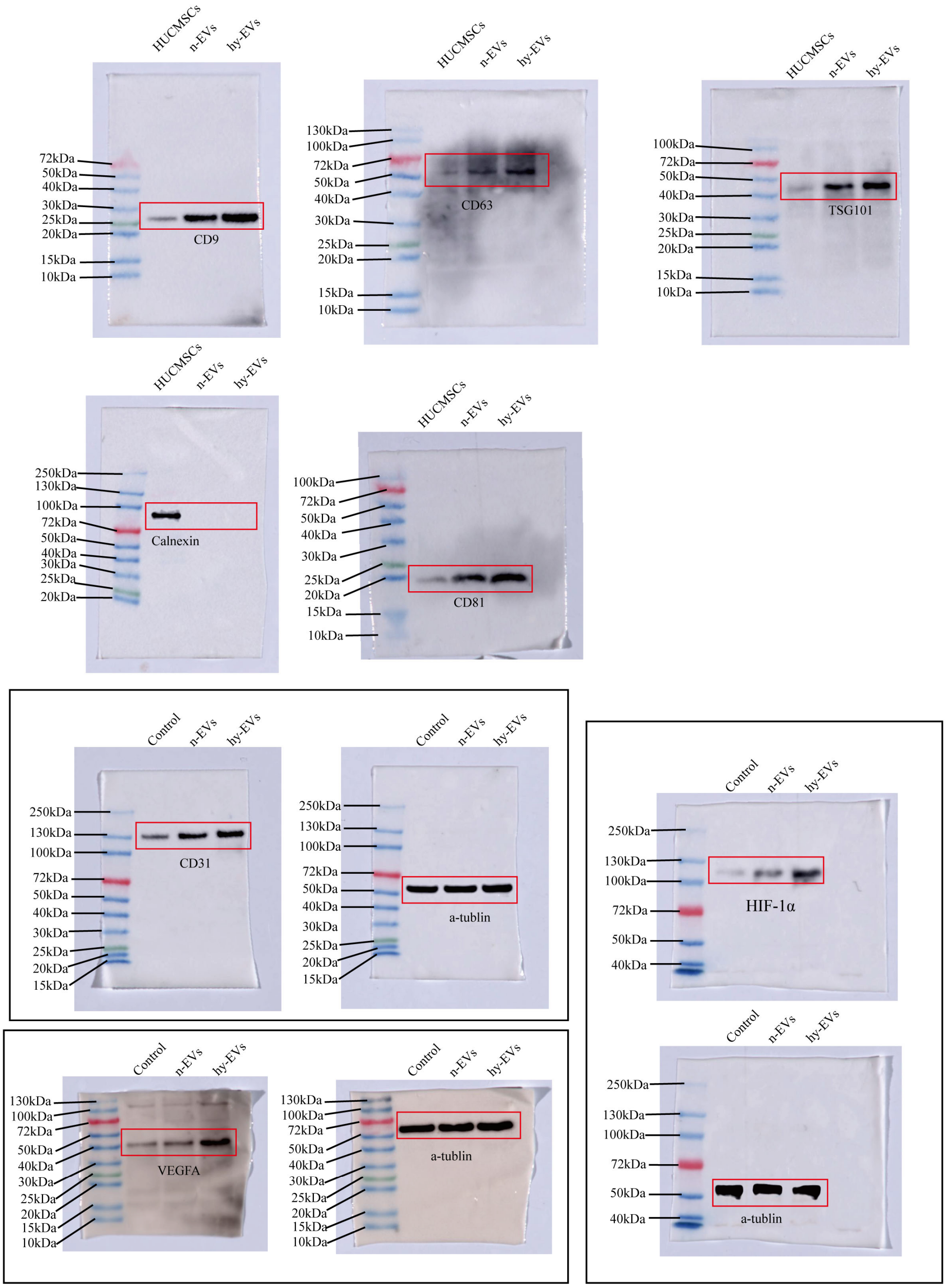

Figure S4. Original Western blot images for Figure 2d and Figure 5g.
